# Family-Oriented Decision Making on Feeding Options: A Case Study of Chinese American Dementia Caregivers

**DOI:** 10.1093/geroni/igaf122.1126

**Published:** 2025-12-31

**Authors:** Zexi Zhou, Jing Wang, Jing Huang, Dena Schulman-Green, Bei Wu, Laura Hanson, Yaolin Pei

**Affiliations:** The University of Texas at Austin, Austin, Texas, United States; University of New Hampshire, Durham, New Hampshire, United States; Johns Hopkins University, Baltimore, Maryland, United States; New York University, New York, New York, United States; NYU Shanghai, Shanghai, China (People’s Republic); The University of North Carolina at Chapel Hill School of Medicine, Chapel Hill, North Carolina, United States; The University of Texas at Austin, Austin, Texas, United States

## Abstract

Making decisions about feeding options, i.e., hand feeding vs. tube feeding, for older adults with advanced dementia is a complex process shaped by cultural values. Decision-making can be particularly challenging for Chinese American families where caregivers must navigate tensions among medical recommendations, cultural norms, and familial obligations. Using a case study approach, this study explores factors that may contribute to informed and effective decision-making about feeding options for loved ones with dementia. Interviews were conducted with three Chinese American family caregivers who had cared for a parent or parent-in-law with dementia for 4–9 years. All three caregivers and their families selected careful hand feeding for their loved ones with dementia. Demographic information and thematic analyses were used to understand their decision-making process. Findings reveal that efficient intra-family communication contributed to timely and well-supported feeding decisions. Achieving consensus among family members (e.g., siblings, adult children) while maintaining family harmony was pivotal. Additionally, when the person with dementia’s feeding preferences were clearly communicated pre-cognitive decline or during lucid moments, decision-making was smoother and more affirmed as compared to proxy decision-making. Access to feeding-related knowledge and effective communication with healthcare providers further facilitated informed and confident decision-making. Findings highlight family orientation as central to decision-making in Chinese American families. Advance care planning with the person with dementia as well as proactive communication with healthcare professionals also play important roles in navigating feeding options. This study supports future culturally sensitive guidance that supports dementia family caregivers to consider unique family dynamics.

